# Advancements in MEMS Micromirror and Microshutter Arrays for Light Transmission Through a Substrate

**DOI:** 10.3390/mi16010103

**Published:** 2025-01-16

**Authors:** Shilby Baby, Mustaqim Siddi Que Iskhandar, Md Kamrul Hasan, Steffen Liebermann, Jiahao Chen, Hasnain Qasim, Shujie Liu, Eslam Farrag, Dennis Löber, Naureen Ahmed, Guilin Xu, Hartmut Hillmer

**Affiliations:** 1 Nanoscale Glasstec GmbH (NaGt), 34132 Kassel, Germany; shilby.baby@nanoscale-glasstec.com (S.B.); mustaqim.iskhandar@nanoscale-glasstec.com (M.S.Q.I.); steffen.liebermann@nanoscale-glasstec.com (S.L.); guilin.xu@nanoscale-glasstec.com (G.X.); 2 Institute of Nanostructure Technologies and Analytics (INA), Technological Electronics Department and Center for Interdisciplinary Nanostructure Science and Technology (CINSaT), University of Kassel, Heinrich-Plett-Straße 40, 34132 Kassel, Germany; kamrul.hasan@ina.uni-kassel.de (M.K.H.); chen@ina.uni-kassel.de (J.C.); qasim@ina.uni-kassel.de (H.Q.); shujie.liu@ina.uni-kassel.de (S.L.); farrag@ina.uni-kassel.de (E.F.); d.loeber@ina.uni-kassel.de (D.L.); naureen.ahmed@ina.uni-kassel.de (N.A.)

**Keywords:** micromirror arrays, light steering, smart window, planarization, closing time, energy saving

## Abstract

This paper reviews and compares electrostatically actuated MEMS (micro-electro-mechanical system) arrays for light modulation and light steering in which transmission through the substrate is required. A comprehensive comparison of the technical achievements of micromirror arrays and microshutter arrays is provided. The main focus of this paper is MEMS micromirror arrays for smart glass in building windows and façades. This technology utilizes millions of miniaturized and actuatable micromirrors on transparent substrates, enabling use with transmissive substrates such as smart windows for personalized daylight steering, energy saving, and heat management in buildings. For the first time, subfield-addressable MEMS micromirror arrays with an area of nearly 1 m^2^ are presented. The recent advancements in MEMS smart glass technology for daylight steering are discussed, focusing on aspects like the switching speed, scalability, transmission, lifetime study, and reliability of micromirror arrays. Finally, simulations demonstrating the potential yearly energy savings for investments in MEMS smart glazing are presented, including a comparison to traditional automated external blind systems in a model office room with definite user interactions throughout the year. Additionally, this platform technology with planarized MEMS elements can be used for laser safety goggles to shield pilots, tram, and bus drivers as well as security personal from laser threats, and is also presented in this paper.

## 1. Introduction

Micro-electro-mechanical systems (MEMSs) are gaining significance today not just in traditional areas like microelectronics and sensor technologies but also in large-scale applications such as displays [1,2], optical switches [3], optical data storage, bar code reading [4,5], scanning confocal microscopy [6], and optical communication [7]. Their increasing integration into these fields is driving innovations that enhance performance and functionality [8] as their properties can be tailored to the needs of diverse applications [9].

The digital micromirror device (DMD) by Texas Instruments (Dallas, TX, USA) is among the most successful MEMS products on the market. This device is widely utilized in portable projectors, large-screen televisions, and digital cinema systems [10]. MEMS-based micromirrors have high operating speeds, a low mass, and the ability to integrate with electronics through batch microfabrication processes, and they are affordable [11]. DMD technology employs switchable aluminum mirrors that can tilt to two stable positions, e.g., +12° or −12°, relative to the substrate normal, representing the on and off states, respectively. The electronic control circuitry corresponding to each pixel is situated below each micromirror. Each DMD pixel is located on a complementary metal–oxide semiconductor (CMOS) circuit board [12].

DMD arrays have opened an amazingly large range of fascinating applications. However, due to the use of silicon substrates (non-transparent in the visible range), the system has to work using reflection (with respect to the substrate). As a consequence, the optical axis is folded, which is a disadvantage for some applications. By using transparent glass substrates for MEMS micromirror or microshutter arrays, additional applications are possible, where the optical axis is not folded. In addition, due to the much lower substrate and technological cost, it also opens possibilities for large-scale MEMS applications such as smart glass for window applications. The goal of this paper is to show what kind of optimizations of distinct technical parameters are possible to address the special needs of specific applications. This paper also describes several trade-offs and counter-running demands concerning the optimization of several parameters at the same time.

As already mentioned, this article addresses a particular field of application of MEMS micromirror or microshutter arrays in transmissive applications, in which the arrays are used to control the light transmission through them via the modulation, guiding/steering, or blocking/shading in a defined direction. Contrary to the common applications with silicon substrates, these technologies are based on a glass substrate, and the light is transmitted through the substrate.

In applications where light modulation and/or light steering through the substrate takes place, distinct differentiations are made based on their main functionality. In this regard, micromirrors and microshutters differ from each other by their main functionality and, consequently, their structure. Micromirrors, with their planarized surface, are able to steer light. Conversely, microshutters are intended for light blocking/shading, so their structure is not planarized—they are often curled or rolled. One transmissive application of micromirror arrays is aimed at the smart glass and switchable glazing sector, which is currently dominated by chromogenic systems, suspended particle device (SPD), and liquid crystal (LC) technologies—all of which are already on the market. Other notable applications are as optical elements in imaging instruments and in laser safety goggles.

The MEMS-based approach using micromirror and microshutter arrays is gradually gaining attraction. Notable breakthroughs in terms of functionality and manufacturing scalability have been achieved since the last reviews on this topic, particularly that of Lamontagne et al. [13] in 2019 and our subsequent update [14] in 2021. These important advancements have brought this technology significantly closer toward commercialization. For this reason, an update of the existing reviews on this topic is appropriate. An overview of MEMS arrays for light modulation through a substrate is provided in Section 2, followed by a presentation of smart window and laser safety goggle applications in Section 3. Subsequently, the recent advancements in MEMS smart glass applications are covered in Section 4. A simulation of the energy-saving potential of MEMS smart windows is presented in Section 5, followed by the conclusion of this paper in Section 6.

## 2. Overview of Technical Achievements in Micromirror and Microshutter Arrays with Light Transmission Through a Substrate in Various Application Fields

MEMSs typically feature a movable component that can be actuated by external forces (like pressure or movement) or internal forces, such as electrostatic and magnetic forces. Their small size contributes to their high sensitivity to these forces while also providing mechanical robustness. The methods for fabricating MEMSs are based on semiconductor manufacturing techniques. While some groups have developed devices using opaque Si wafers, most efforts have focused on controlling light transmission through switchable glass in smaller applications, like spectrometer slits and eyeglasses, as well as larger settings, such as car sunroofs, aircraft windows, and building windows [13,14]. MEMS devices fabricated on silicon substrates can only be used in reflection applications, whereas MEMS devices fabricated on glass substrates can be used in transmission and reflection applications.

NASA developed a microshutter array using MEMS-based transmissive slit masks for the Near Infrared Spectrograph (NIRSpec) instrument on the James Webb Space Telescope (JWST). These microshutters were engineered for the selective transmission of light, ensuring high efficiency and contrast [15]. On the other hand, spectrometers equipped with digital micromirror devices (DMDs) from Texas Instruments perform object selection by tilting the micromirror between its two stable positions. Here, the light is either reflected toward the spectrometer or out of the optical path. However, its minimum operating temperature is −40 °C, making it unsuitable for IR observations.

Canonica et al. [16] presented a two-dimensional monocrystalline silicon micromirror array as a field selector for astronomical spectroscopy. Object selection is accomplished by precisely tilting micromirrors, which direct the light from objects toward the spectrometer. When the micromirror is at rest or not tilted, the light from the astronomical objects and the sky background are reflected back to space. This device has an operating temperature that ranges from room temperature to cryogenic temperature (<100 K) [16].

Lamontagne et al. [13] published a review article on microshutters fabricated on glass substrates to control the light transmission, giving a valuable overview of the technology. An updated version of the comparison table from Lamontagne et al. to include the recent values is presented in Table 1.

Nanoscale Glasstec GmbH (NaGt) produced a MEMS smart glass with an area of nearly 1 m^2^ with 28 individually addressable subfields. The University of Kassel fabricated microshutters with planarized metallic blades with a closing time of 1 µs [17,18] and a modulation contrast between the open and closed status of 7700 [14].

The Microelectronic Center of North Carolina (MCNC) reported a microshutter designed to protect optical sensors. This device features a transparent substrate, a transparent conducting electrode, insulating polymers, and a reflective top electrode layer. The devices operate at a high voltage of approximately 100–300 V, with a closing speed of 18 μs. They found that lower operating voltages lead to longer lifetimes, having tested the devices for up to 450 million cycles [19,20].

Microshutters from the National Research Council (NRC) Canada for smart window applications have a low operating voltage of around 20 V and a high contrast ratio of T_max_/T_min_ of 600 [13].

The New Visual Media Group (NVMG) uses shrinkable thin metalized polymer foil to make electropolymeric shutters for windows and displays. When voltage is applied, electrostatic forces cause the shade to roll down, and, conversely, they roll back when voltage is removed. Their shutter size is in the order of millimeters. They have a high actuation voltage of up to 500 V and are available commercially with a larger area [21].

The Institut National d’Optique (INO) utilizes microshutters for spectrometry applications. Each shutter regulates the light that passes through a microslit defined on the transparent substrate that supports the array. A pull-in voltage of approximately 110 V closes the microslit, with response times of about 2 ms to close and 7 ms to open [22].

The United States Air Force developed microshutters for adaptive coded aperture imaging and non-imaging applications. These shutters employ corrugations to enhance the curling mechanism but also encounter challenges in releasing, which varies based on the geometry of the microshutters. They have a contrast ratio of T_max_/T_min_ of 40 [23].

**Table 1:** Comparison of diverse MEMS shutter technologies concerning materials, size of single shutter elements, experimental characterization results, and potential applications. This table serves as an update to the recent publications [13,14,17] on this topic.

Research Group	Bottom Electrode	Top Electrode	Size	Actuation Voltage	Closing Speed	Demo. Size	T_max_/T_min_ Contrast	Applications, Remarks
Fiat [1,2]	ITO	Flexible metal layer	458 µm to 2.4 mm	20–100 V	0.1 ms	-	20/1 = 20	Automotive display, IR spectrometry, low transmission
MCNC [19]	ITO	Polyimide/Cr/ Au/polyimide	<100 µm to over 200 µm	100–300 V	18 μs	5 cm^2^	Low contrast	Eyelid for protection
NaGt, University of Kassel [14,24,25,26,27]	FTO, ITO, Ag low-e	Al, Cr, Ge	150 × 400 µm^2^	12–80 V	0.1 s	9100 cm^2^	73/0.01 = 7300 High contrast	Sunlight steering for buildings; high contrast, low voltage
University of Kassel [17,18]	FTO, ITO	Al, Cr	60 × 1000 µm^2^ 100 × 1000 µm^2^	50–80 V	1 µs	12 cm^2^	77/0.01 = 7700 High contrast	Laser safety goggles; high contrast, high closing speed
NRC [13]	SnO_2_, ITO, Ag low-e	Cr and others	50–300 µm^2^	15–25 V	40 µs	20 cm^2^	60/0.1 = 600 High contrast	high contrast, low voltage
NVMG [21]	TCO	Shrinkable polymer	≥2 mm	100–500 V	seconds	5000 cm^2^	Low contrast	Macro-curling shutter, commercialized
INO [28,29]	Al	MoCr	60 × 1000 µm^2^	110 V	2 ms	0.25 cm^2^	Low contrast	Space instrumentation
Air Force [23]	AlZnO	Ti and Au	200 × 50 µm^2^	-	-	-	40/1=40	Adaptive coded aperture imaging
Samsung [30,31]	ITO	Al-SiNx, Mo-Mo	ᴓ 2.2 mm, 36 × 1.4 mm long triangular rolled shutters	30 V	2 ms	Iris of 0.04 cm^2^	-	Iris shutter for camera
KAIST [32,33]	ITO	Electroplated Ni	200 × 160 µm^2^	20–30 V	20 µs	Small	T_max_ = 60	Active transparent display with TR-OLEDs
Tokyo University [34]	ITO	Al-SiO_2_	200 × 30 µm^2^	38–55 V	3 ms	-	53/36 = 1.5	Implemented on TFT
Stuttgart University [28]	MoTa	MoTa on stressed SiNx	200 µm	20–60 V	-	2–225 cm^2^	Low contrast	Transmissive display on TFT, low transmission
TU Beijing [35,36]	-	-	5 × 2.5 mm^2^	2.8–8 V	14 ms	400 mm^2^	63.3/3.6 = 17.5	Electrothermally actuated micro-shutters, automobile headlights

Samsung (Seoul, Republic of Korea) introduced an iris shutter with a 2 mm diameter for cameras using MEMS microshutters. The iris consists of 36 individual triangular shutters, each measuring 1.4 mm in length. The fabricated shutter has a closing time of 2 ms with an actuation voltage of 30 V [30,31].

The Korea Advanced Institute of Science and Technology (KAIST) built cascaded microshutters for variable transmission to enhance the light efficiency of transparent organic light-emitting diode displays. This microshutter device operates with a control signal of 20 V to 33 V. The switching time is 20 µs when transitioning from the open state to the closed state, with an estimated lifetime of 500 billion cycles [32,33].

The University of Tokyo fabricated electrostatically reconfigurable roll-up microshutters utilizing a stress gradient based on evaporated metal on SiO_2_ at 180 °C. Each individual shutter measures 200 μm in length, 30 μm in width, and 0.3 μm in thickness. The shutter is electrostatically closed at 55 V, and the closing speed is approximately 3 ms. They have very low contrast, with an optical transmittance of 36% for the closed position and 53% for the open position [34].

Stuttgart University developed a MEMS shutter display for transmissive display applications. They are fabricated on thin-film transistor (TFT)-based active matrices with a wet-etched Si sacrificial layer 150 to 200 nm thick. They have low contrast, but the operating voltage is around 30 V, and the estimated response time is below 50 μs [28].

Unlike the transparent electrodes and glass substrates used for optical transparency in the previously mentioned studies, the Beijing Institute of Technology introduced microshutters on a silicon substrate for analog light control. This design features a through-silicon cavity beneath the bimorph to facilitate light transmission. This bimorph comprises two layers—aluminum (Al) and silicon dioxide (SiO_2_)—which are materials with different coefficients of thermal expansion (CTEs). At room temperature, the residual stresses in these two layers cause the bimorph to curl, resulting in an open state. The temperature of the bimorph can be adjusted by altering the voltage applied to the embedded titanium (Ti) or platinum (Pt) heater. When the temperature rises, the radius of the curvature of the bimorph increases, and, at a certain value, the bimorph becomes flat (closed state). Even though they have low driving voltage of 3 V, the power consumption is quite high at 9.1 mW/mm^2^ [35,36].

Most research on microshutters has focused on devices covering small areas. However, the electrostatic principle used for operation can be scaled to larger areas, as demonstrated by NVMG. Comparing the speed and operating voltage of different methods is complex since it is influenced by the sample size and the geometry of the metal grid, including the metallic stripes. Recently, we conducted theoretical model calculations that accounted for the electromechanical closing and dynamic signal transport propagation for the micromirror located farthest from the array’s edge. We observed a nonlinear relationship between the closing time and stripe length for the TCO/SiO_2_/metal stack and 3D stripline geometry. Consequently, we believe that all groups listed in Table 1—with the exception of NVGM—conducted measurements on small samples or near the array’s edge, which resulted in similar findings due to the small sample sizes [17].

The technical achievements of the twelve research groups can be compared as follows:

- Table 1 includes the work of eleven research groups developing microshutter arrays and one research group developing micromirror arrays.
- The transmission of all twelve MEMS arrays works through a transparent substrate, and they cover applications such as displays, eyelid protection, and safety goggles, smart glass in buildings, space instrumentation, imaging, iris shutters, and automotive lighting.
- Electrostatic actuation provides a much faster temporal response than electrothermal actuation.
- One group used electrothermal actuation [32,33], while the other research groups used electrostatic actuation—most probably due to the lower power consumption and faster actuation speed that could be obtained.
- Smart glass applications for buildings were envisaged by [2,11,14,19,20,24,25,26,37,38,39].
- Only one research group (dealing with micromirror arrays) developed stress-compensated planar mirrors. Effective light steering is only viable with planar mirrors. Thus, the rest of the eleven methods are categorized as microshutter array concepts allowing light modulation, whereas the micromirror array concept achieves light modulation and light steering.
- The remaining eleven research groups used the unrolling and up-rolling of curled shutter blades. In case of the maximum transmission (open state), all the shutters in a row along the hinge direction form a roll. In that case, the light transmission through the glass substrate is shaded by half of a roll and the anchor where the roll is attached. In contrast, the micromirrors in the open state can stay in the shadow of the anchor and do not contribute to reducing the maximum transmission. Therefore, the planar mirrors enable the highest potential for maximum transmission in the open state.
- The microshutter and micromirror geometries are mainly rectangular, with two exceptions using triangular [30,31] as well as triangular and pentagonal shapes [40]. The two latter geometries allow not only rectangular array shapes but also free-form array shapes including circular and ring ones.
- Scaling up the array areas was only performed by two groups [14,21], reaching areas of 5000 cm^2^ and 9100 cm^2^, respectively.
- Considering the lowest actuation voltage, only thermal actuation can deliver voltages below 3 V, provided that the application scenario does not require fast actuation at the same time. The lowest actuation voltages achieved with electrostatic actuation are considerably higher and have been demonstrated by two groups (12 V and 15 V) [13,14]. All the other electrostatic voltages are even much higher and can go beyond 100 V.
- In the experiments, a trade-off between low actuation voltages and short actuation times was observed. At the expense of much longer actuation times, very small actuation voltages can only be achieved via electrothermal actuation. Conversely, this means that very short actuation times are only possible at the expense of higher actuation voltages. In addition, note that the fastest reported closing time of 1 µs could only be obtained on small MEMS array areas and for voltages of 80 V or higher.
- As expected, the actuation times (e.g., from fully open to fully closed) are related to the array size. This is because the actuation times are based on the electro-mechanical switching time of a single mirror, the delay times of strip lines, and the RC (resistance and capacitance product). The capacitance is strongly area-dependent. For a 12 cm^2^ area, a closing time of 1 µs was obtained, and, for 9100 cm^2^, the closing time was measured as 100 ms. As very short actuation times and large areas are opposing demands, they cannot be obtained at the same time.
- Low electrostatic actuation voltages and short actuation times are also opposing demands: they cannot be obtained at the same time.

This shows that for transparent substrates, the technical parameters, geometries, and sizes of the MEMS blades in the arrays can be individually optimized for various applications. Nevertheless, certain limits are to be expected in cases where opposing demands are involved, and, therefore, trade-offs have to be considered.

## 3. Micromirror and Microshutter Arrays as Smart Windows and Laser Safety Goggles

The DMD arrays mentioned in Section 1 are fabricated on silicon substrates. This technology cannot be applied in, e.g., smart building windows. Silicon wafers are today not available for areas larger than 0.16 m^2^, the technology would be too expensive on a large scale, and transmission through the substrate is definitively required. Therefore, a silicon platform cannot be used in smart glass for buildings: glass needs to be used as a substrate to achieve light transmission through the substrate. A platform technology based on micromirror arrays has been developed in our laboratory based on transparent glass substrates for application in smart windows, laser safety goggles, microscopes, and endoscopes.

**Figure 1:**
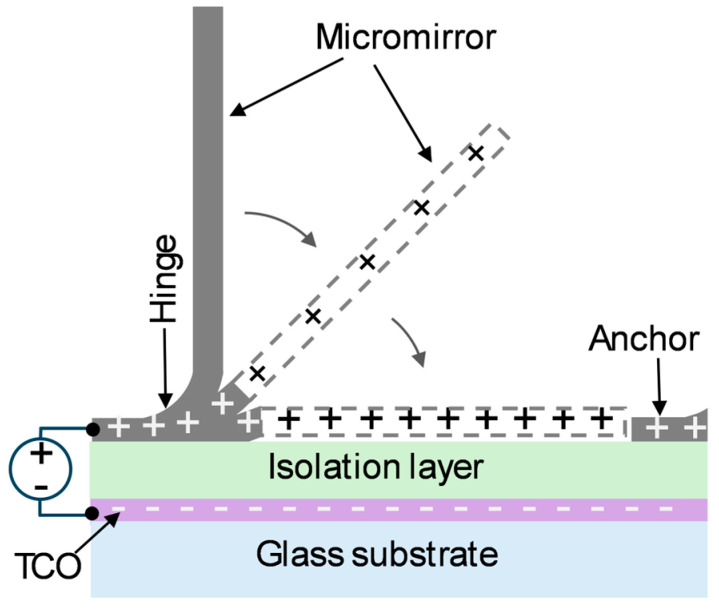
Illustration of the electrostatic actuation concept of a single micromirror.

Typically, the arrangement consists of a glass substrate with a transparent conducting oxide (TCO) layer as the bottom electrode and metallic micromirrors as the top electrode, with an isolation layer in between to separate the electrodes. A single micromirror has three essential components: a fixed anchor, a bent hinge, and a flat mirror with one side fixed, like a hinged door. The micromirror is constructed from a multilayer metal stack consisting of aluminum (Al) and chromium (Cr). The multilayer stack combination and individual layer thicknesses are varied to achieve residual stress at the hinge area and planarized mirror area, thus resulting in a freestanding micromirror at an angle 90° to the substrate (Figure 1).

When a potential difference is applied between the mirror plane and the counter bottom electrode, the micromirror approaches the substrate and can be held at various intermediate angles (tilt angle Φ) by balancing the electrostatic attraction force and counteracting the restoring force resulting from the residual stress prior to the critical pull-in point. When the micromirror exceeds the critical pull-in angle, the micromirror snaps horizontal to the substrate due to the pull-in effect. This mechanism allows dynamic light steering toward the ceiling through the active control of the reflection on the micromirror surface. The angular movement is governed by the interplay between the elastic restoring force and the electrostatic attraction force, in which the pull-in phenomenon is involved. A detailed semi-analytic work on the bending movements of similar micro-cantilever structures can be found in the paper of Sokoluk et al. [41].

Electrostatic MEMS micromirrors are especially appealing due to their efficiency, which includes high energy density and significant force output, as well as their straightforward design, rapid response times, and low power consumption [9,13,14]. This electrostatically actuatable micromirror array serves as a versatile platform technology, and, by altering the shape, thickness, and dimensions of the micromirrors, it can be adapted for various applications. Two notable uses of these micromirror arrays are as MEMS smart windows for personalized light steering and as microshutter arrays for laser safety goggles. To avoid overextending, these two applications are the focus of this paper.

### 3.1. MEMS Smart Window for Personalized Light Steering

CO_2_ emissions are one of the major causes of global warming. It is estimated that doubling the atmospheric CO_2_ concentration leads to a temperature increase of about 5–6 °C, according to climate models [42]. The building sector accounts for approximately one-third of all raw materials and energy consumed in Europe, as well as 35% of the carbon dioxide emissions, contributing significantly toward global warming and climatic changes. Moreover, a significant portion of the building stock is energy-inefficient, with only a small percentage undergoing renovations annually [37]. According to the “Building Report KOMPAKT 2019” by the German Energy Agency, space and water heating as well as lighting are the main contributors to the energy consumption of buildings [43].

The concept of “Smart Green Buildings” has recently garnered considerable interest, highlighting the need to integrate energy-efficient equipment into all buildings and to enhance the use of renewable energy sources such as daylight for lighting, heating, and cooling [44]. For instance, among the various recent studies, the “Light and Health” investigation conducted in 1990 demonstrates that workers located near windows in commercial buildings face the lowest health risks as they experience less fatigue, dizziness, job dissatisfaction, depression, tension, and disruptions to their natural working rhythm [27]. Windows and glazing are essential elements of buildings, playing a vital role in effective insulation by reducing heat loss and capturing or reflecting solar heat depending on the requirement. Windows also offer access to natural light, ventilation, and enjoyable views for the room occupants. However, only the regions near the windows are illuminated efficiently, whereas the remaining area of the room might suffer from insufficient lighting, thus requiring artificial sources to provide sufficient ambient lighting inside the room. Moreover, a low angle of sunlight incidence in the morning and evening, as well as during the winter season, can cause uncomfortable glare effects on computer screens or displays, thereby disturbing the occupants near the window considerably [45,46,47,48]. Additionally, a large proportion of glazing globally is not energy-efficient, often consisting of single glazing or older double glazing without low-emissivity (low-e) coatings [38]. Windows are frequently viewed as the least energy-efficient part of a building, responsible for approximately 60% of the total energy loss, which occurs through heat conduction, convection, and radiation. This inefficiency leads to higher energy demands for lighting, heating, and cooling [49].

**Figure 2:**
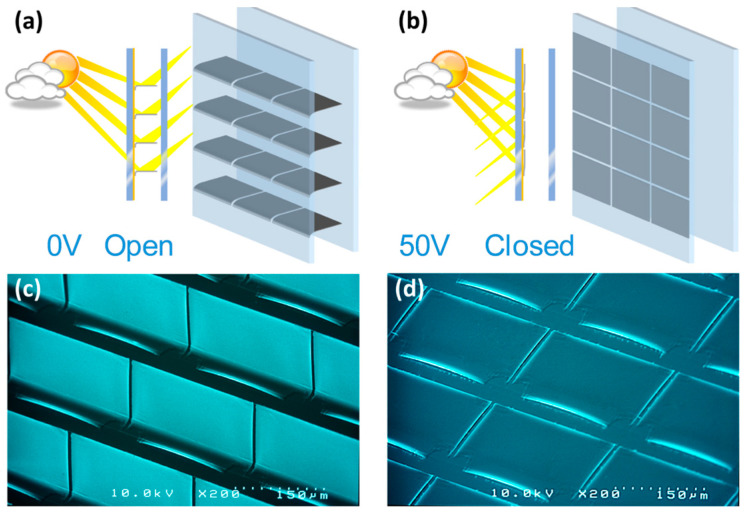
Schematic diagram of a micromirror array in (**a**) the open state at 0 V and (**b**) the closed state at 50 V. They are arranged in an insulated double-glazed windowpane with an inert gas atmosphere inside and sealed with butyl. Their SEM micrographs of both open and closed states are shown in (**c**) and (**d**), respectively. Modified Figure from [27].

To address these limitations, various smart window products are currently available on the commercial market. These include passive systems such as light shelves, window blinds, photochromic, and thermochromic systems, as well as active window systems utilizing suspended particle devices (SPDs), electrochromic systems, and PDLC. Additionally, a distinct approach to intelligent glazing using MEMS-based micromirror arrays has been developed featuring millions of electrostatically actuatable MEMS-based micromirrors inside a double-glazed windowpane (Figure 2) in an inert gas (e.g., Ar or Kr) environment, enabling enhanced functionality such as daylight blocking for security and privacy, effective daylight distribution, and personalized lighting ambiance [39].

The micromirror arrays embedded in a double- or triple-glazed windowpane are shielded from dust, strong winds, cleaning processes, and harsh weather conditions, enhancing their longevity and overall stability. These tiny micromirrors are nearly invisible to the plain sight from distances greater than 25 cm, and they offer a long lifespan, high stability, low power consumption, significant energy-saving potential, cost-effectiveness, fast switching speed, and a wide operating temperature range [24].

It is important to mention that the envisioned operation of micromirror arrays as smart windows requires a complete control circuit in order to exploit their full potential. This includes the integration of a driving circuit and various sensors, such as for the detection of user position and sun position, which are commercially available. The topic of the actuation system and its related characterization is another independent topic and shall be published later in a separate article.

**Figure 3:**
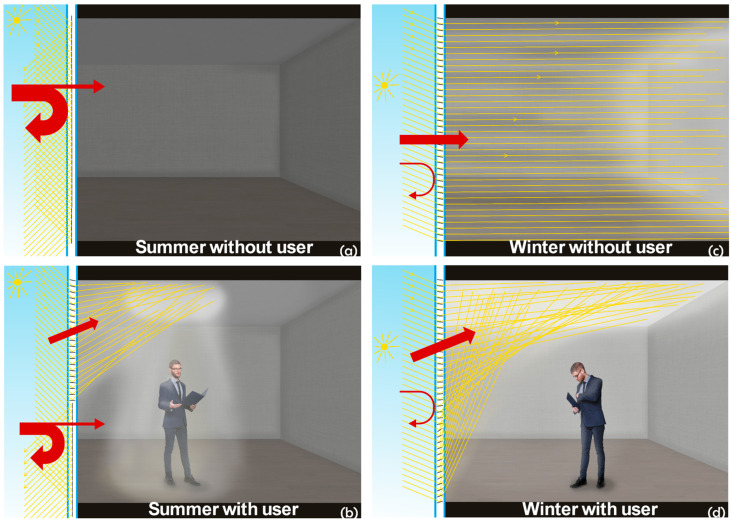
Illustration of a room in which smart micromirror-array-based windows have been installed in summer and winter scenarios with and without users, demonstrating light steering and heat energy management. (**a**) Summer, user absent: blocks solar radiation by reflecting to the outside, keeping the room cool. (**b**) Summer, user present: illuminates the room by deflecting light toward the ceiling above the user, thus saving the energy used for cooling by limiting the heat transfer with closed micromirrors in the lower areas. (**c**) Winter, user absent: acts as a radiation heater by using all solar infrared and visible radiation to heat the room. (**d**) Winter, user present: reflects the complete solar radiation toward the ceiling while minimizing glare. Heat radiation coming inside the room is represented using red arrows, whose width symbolizes the amount of heat energy entering the room or being reflected. Original figure from [27]; reprinted with permission of Leuze Publishing House.

Based on factors such as window orientation, building latitude, season, time of day, and user presence, many scenarios can be created. Four of these scenarios are illustrated in Figure 3. Micromirrors, in their open state, enable daylight steering by reflecting light toward the ceiling or toward a wall by adjusting their opening angle. On the other hand, micromirrors, in their fully closed positions, completely block the solar radiation from the outside.

During summer, when sunlight intensity is high, and no users are present in the room, all micromirrors can be switched to a closed position, effectively minimizing heat transfer and keeping the room cool (Figure 3a). An automated control unit utilizes data from an intelligent sensing network that monitors factors like person location, sun position, indoor temperature, and ambient light levels to optimize performance based on specific needs.

When user presence is detected in summer, some of the micromirrors in the upper areas (Figure 3b) remain open to reflect light toward the ceiling above the user, while the micromirrors in the lower part of the window stay closed to keep the unoccupied areas of the room cool. This selective opening and closing of the micromirrors allows for significant energy savings by reducing heat transfer into the room. Additionally, the illuminated spot on the ceiling can follow the user, allowing for efficient lighting even in areas far from the window.

During winter, when no users are detected in the room, all micromirrors remain open to maximize solar energy capture, effectively using the available solar radiation to warm the space. This approach acts like a radiator, significantly reducing the energy needed for heating (Figure 3c).

Conversely, when user presence is detected in winter, the micromirrors are kept open to direct solar radiation toward the ceiling but at varying angles using tailored actuation voltages to minimize glare. This strategy not only enhances lighting but also conserves the energy needed for both heating and illumination (Figure 3d).

### 3.2. MEMS Microshutter Arrays for Laser Safety Goggles

Since 2005, the number of reported laser attacks from ground-based individuals targeting arriving and departing aircraft has surged by over 300%. A laser directed at an aircraft windshield can startle pilots and potentially cause injury. When high-power laser beams enter the cockpit of a plane or helicopter, it can damage the pilot’s eyes or temporarily lead to glare effects, making it challenging to operate or land the aircraft safely [50].

Conventional passive goggles can only absorb specific wavelength ranges, leading to a coloring effect when designed for visible laser wavelengths, as some wavelengths are filtered out of the white light spectrum. In traffic applications, such as for pilots and other drivers, passive laser goggles are unsuitable due to their low transmission during night-time when most attacks occur and the inability of users to accurately perceive the colors of displays or critical indicators. Each wavelength that passive goggles block complicates the multilayer coatings, reducing overall light transmission (down to 35%) and negatively affecting color recognition. Reports of laser attacks against pilots have involved various wavelengths, including green (532 nm), red (640 nm and 650 nm), and blue (405 nm), further highlighting the inadequacy of passive protection [17].

**Figure 4:**
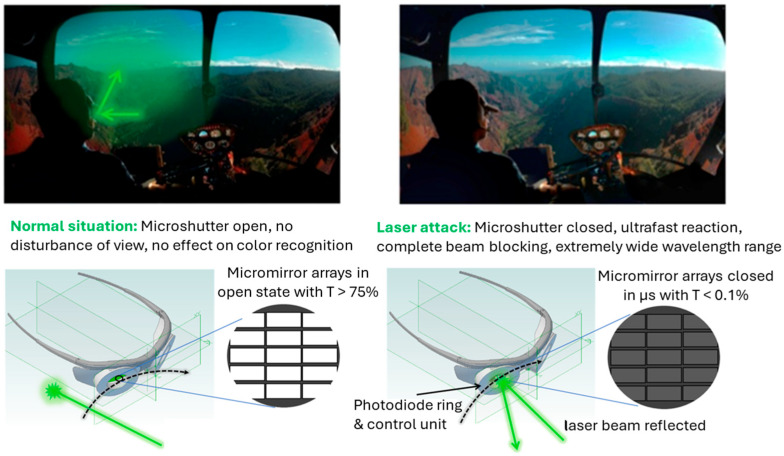
Illustration of the working principle of MEMS-based laser safety goggles when used by a pilot during normal situations (**left**) and a laser attack (**right**).

These challenges have driven the development of MEMS microshutter arrays for laser safety goggles (Figure 4), incorporating advanced sensing and control technology capable of blocking laser light across any wavelength that exceeds defined eye safety levels.

Active laser safety goggles are designed to protect against potential laser attacks targeting pilots, tram and bus drivers, police, and security personnel. The ultrafast closing of MEMS shutters can achieve this once the integrated photodiodes detect the incoming laser radiation [17]. Compared to micromirrors for light steering, microshutters for laser safety goggles do not require an intermediate state. They are either completely open or completely closed. Important conditions for laser safety goggles are maximum transmission in the open state, minimum transmission in the closed state, and fast switching speed. These can be achieved by modifying the dimensions of the micromirrors, substrate thickness, and the type of TCO used.

The metallic MEMS microshutter arrays in laser safety goggles remain in a fully open state, allowing light to pass through to the lenses and reach the eyes under normal conditions. These shutters are arranged in an array connected by a grid, enabling them to move synchronously with their neighboring shutters. In the event of a laser attack, the shutters close rapidly to completely block incoming light of all wavelengths, preventing serious eye damage from laser exposure. Once the laser radiation has ceased, the shutters reopen, restoring clear visibility for the pilot [17]. The signal for fast closing and reopening is generated by a photodiode-ring surrounding each goggle glass.

The metallic MEMS microshutter arrays in laser safety goggles remain in a fully open state, allowing light to pass through to the lenses and reach the eyes under normal conditions. These shutters are arranged in an array connected by a grid, enabling them to move synchronously with their neighboring shutters. In the event of a laser attack, the shutters close rapidly to completely block incoming light of all wavelengths, preventing serious eye damage from laser exposure. Once the laser radiation has ceased, the shutters reopen, restoring clear visibility for the pilot [17]. The signal for fast closing and reopening is generated by a photodiode ring surrounding each goggle glass.

These active laser goggles not only address the issues mentioned above but also meet high safety, security, and health standards. In summary, MEMS microshutter array goggles are color-neutral, offer much higher light transmission, provide significantly greater optical density (OD) in the closed position compared to most passive laser goggles, and offer significantly higher protection. Conventional passive goggles can only absorb specific wavelength ranges, leading to a coloring effect when designed for visible laser wavelengths, as some wavelengths are filtered out of the white light spectrum. Additionally, a single scratch on the coatings of passive goggles can compromise their protective capabilities [17].

## 4. Current Achievements in MEMS Smart Glass Based on Micromirror Arrays

An optimal daylighting system should ensure the maximum utilization and uniform distribution of daylight while being flexible enough to meet varying lighting needs. While reductions in cooling, heating, and lighting loads with low power consumption are the main goals for energy-efficiency purposes, having clear visibility, high switching speeds, and low maintenance costs are also essential for day-to-day use. Furthermore, cost-effective manufacturing, installation, and operation are fundamental to commercial viability. An effective micromirror design must achieve a balance between various competing factors, including excellent uniformity in layer thicknesses and compositions, high material purity to prevent unwanted absorption, a low minimum transmission (T_min_) in the closed state, a high maximum transmission (T_max_) in the open state, quick switching speed, low actuation voltage, low haze, low-temperature sensitivity, long lifespan, and minimal sensitivity to solar UV radiation.

At present, the smart window technologies that are available on the market only meet these requirements to a limited extent [24]. Among these, the micromirror-array-based window is considered as one of the most promising solutions for existing buildings thanks to its lower manufacturing costs and significant energy-saving potential. Some of the recent achievements in MEMS micromirror technology are described in this section. Since some of these parameters are contradictory (e.g., switching speed and actuation voltage), certain results have been obtained from different samples.

### 4.1. Free-Standing Planar Micromirrors

**Figure 5:**
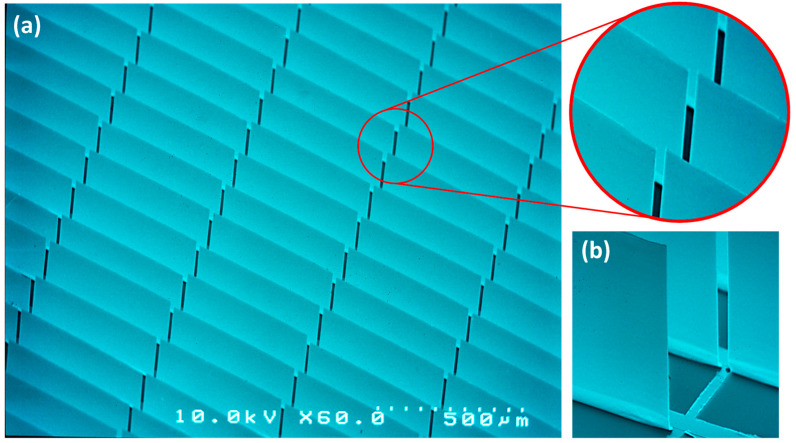
SEM micrograph of (**a**) free-standing micromirror arrays after the lift-off and drying process with an inset of the magnified area, (**b**) individual vertical standing flat mirror with an ~90° opening angle. Original figure from [27], reprinted with permission of Leuze Publishing House.

Due to the resulting residual stress in the thin metal layers responsible for the opening of micromirrors, fabricated micromirrors are curled. While curled micromirrors enable light modulation and function primarily as a daylight shading system, they do not provide efficient light steering. Moreover, curled micromirrors block a larger part of the transparent area, limiting the maximum transmission during their open state. Therefore, it is essential to planarize micromirrors to achieve efficient light steering and maximize transmission in the open state. The planarization of the micromirror plane is achieved via localized stress compensation. Figure 5 shows an SEM micrograph of the planarized micromirror array fabricated via localized stress compensation.

The fabrication process of both micromirror and microshutter arrays involves typical surface micromachining techniques, which have also been described in previous publications [14,39]. The structuring of the FTO layer is executed via an acid–metal etch process, followed by the deposition of a 1 µm SiO_2_ layer using a plasma-enhanced chemical vapor deposition (PECVD) system (Plasmalab 80 by Oxford Instruments, Bristol, UK). The optical lithography process is executed to define the micromirror design and structure, utilizing the spin-coating method for the photoresist coating and a mask aligner system (MA6 by SUSS MicroTec, Garching, Germany) for the UV exposure. The metal stack layer is deposited using an e-beam physical vapor deposition (EBPVD) system (PLS 500 by Pfeiffer Vacuum, Asslar, Germany), and the structures are then released with standard lift-off and drying processes.

Typically, in most of our designs, the micromirror layer (metal stack layer) consisting primarily of Al and Cr is deposited with thickness featuring 60 to 200 nm of Al and 10 to 40 nm of Cr. These parameters can vary based on the design, the type of deposition process, the deposition rate, and the desired opening angle of the micromirror. As a platform technology with various applications and hence different requirements, there is no single fit-for-all layer system. All designs and any additional materials, such as Ge for coloring and antiglare functioning in a separate work [25], are tailored for the specified requirement and require the fine tuning of the layer stack thickness and arrangement.

### 4.2. Actuation Voltage and Actuation Speed

The actuation voltage of a micromirror array is the voltage required to fully close the micromirrors. It depends on various factors like the metal layer stack, the transparent bottom electrode, and the isolation layer. A typical micromirror for smart window application has thicknesses of between 10 nm and 100 nm in the vertical direction and mirror sizes of typically 150 × 400 μm^2^ in the lateral direction. The electrostatic actuation voltage required to fully close micromirrors is generally between 40 V and 80 V. The optimization of the micromirror design by reducing the length of the hinge has significantly lowered the actuation voltage by more than half. The use of broken hinge micromirrors improves the overall actuation characteristics, and an actuation voltage as low as 12 V was measured.

**Figure 6:**
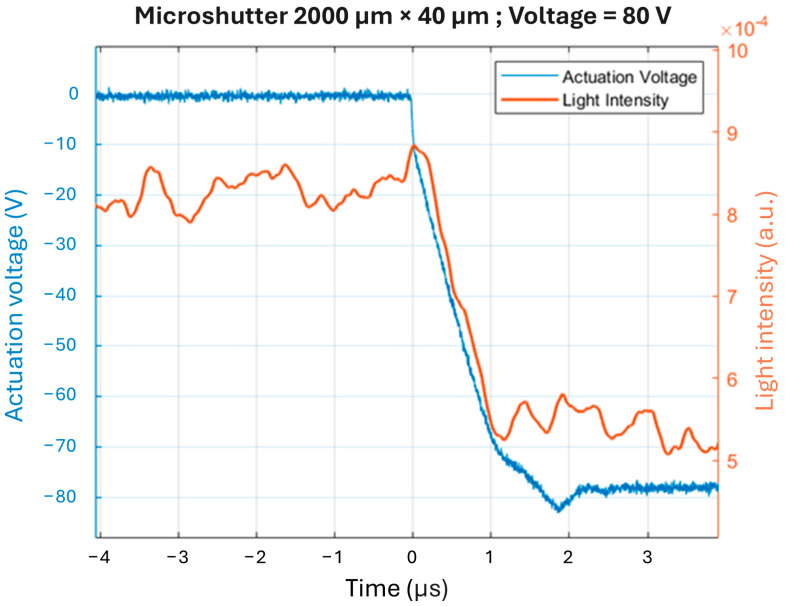
Light intensity transmitted through a microshutter array and the applied electrostatic actuation voltage as a function of time. A closing time of less than 1 μs was measured for microshutter arrays with mirror dimensions of 40 μm × 2000 μm. Original figure from [17].

One of the advantages of the MEMS micromirror is its short switching time between its open and closed state. It takes less than 0.1 s for micromirrors to close once the actuation voltage is applied between the micromirror (top electrode) and counter bottom electrode (TCO). However, a faster closing time is necessary for laser safety goggles to protect the eyes from laser attacks. Simulations (using COMSOL Multiphysics^®^ 5.6) indicate that reducing the microshutter dimensions perpendicular to the hinge decreases the closing time [51]. However, the actuation voltage also influences the closing time of microshutter arrays. Therefore, a compromise must be made between a low actuation voltage and a fast closing speed. The shutters can be optimized for ultra-fast closing at high voltages or for low actuation voltages with a tolerable closing speed. The highest switching speed measured was for a microshutter with dimensions of 40 μm × 2000 μm, which had a closing time of about 1 μs for an actuation voltage of 80 V [17]. Figure 6 illustrates the applied electrostatic actuation voltage and the light intensity transmitted through a microshutter array as a function of time.

Regarding speed, conventional blinds can take about 10 s for a complete open-to-closed transition, with adjustments completed in approximately 1 s for a window unit measuring 1.69 m^2^. In contrast, MEMS smart glazing operates much faster, taking less than 0.1 s, and is independent of temperature. Other technologies exhibit significant temperature dependence in their functionality, particularly in the switching speed. Electrochromic technologies exhibit the slowest switching speed, taking up to 1 h at 0 °C, while SPD technology has the fastest speed of 0.1 s at 20 °C. Thus, MEMS technology excels in this area. Notably, it is important to note again that switching speed is also significantly affected by the sample size [17].

### 4.3. Optical Transmission

For transmission applications like smart windows and laser safety goggles, high optical transmission in the open state and low transmission in the closed state are necessary. For MEMS smart micromirror-glazing, a minimum transmission (T_min_) of 0.01% and a maximum transmission (T_max_) of 73% were measured, including with a glass substrate and a TCO layer. This resulted in a modulation contrast (T_max_/T_min_) of 7300 between the open and closed states. The maximum transmission can be improved by planarization as well as increasing the micromirror dimensions parallel to the hinge. A sample of a MEMS microshutter array with optimized dimensions exhibited a minimum transmission (T_min_) of 0.01% and a maximum transmission (T_max_) of 77%, including the glass substrate with a TCO layer. This resulted in a modulation contrast (T_max_/T_min_) of 7700 between the open and closed states [17].

In terms of transmission, conventional blinds achieve a maximum contrast of about 1000 between their maximum and minimum transmittance. Other technologies, such as electrochromic systems and SPD technology, demonstrate significantly lower values, with contrasts of 60 and 120, respectively. Regarding color neutrality, electrochromic technologies yield the least color-neutral results, whereas conventional blinds and MEMS smart glazing provide the best color neutrality.

### 4.4. Scalability to Typical Window Size Range

Most research on micromirrors has focused on devices with limited size coverage. Nevertheless, the scalability of flat-panel displays (FPDs) using LCD technology, which has functionality akin to micromirrors but involves more complex electronics, is promising. These LCD devices can be produced on glass panels that are more than 4 m^2^ in size and require numerous high-resolution patterning steps [52,53,54]. In contrast, large-area micromirror array panels are less complex than LCD panels and can be manufactured using similar production processes.

**Figure 7:**
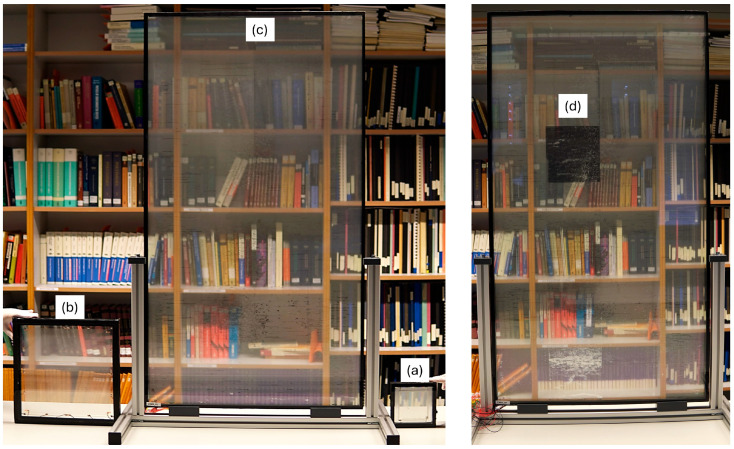
Image of micromirror array modules housed as double insulation glazing using standard window module manufacturing process. (**a**) Initial laboratory-sized module, 10 × 10 cm^2^; (**b**) 30 × 30 cm^2^ module from the intermediate step in upscaling to a larger size; and (**c**) recent achievement of a large-scale module of 71 × 128 cm^2^, intended for a window size of about 80 × 140 cm^2^ with a full frame. (**d**) Actuation of a single subfield from the large-scale 71 × 128 cm^2^ module.

Recently, our group successfully fabricated micromirror arrays on large substrates of 71 × 128 cm^2^ (Figure 7) using a very-low-cost fabrication process—considering such large-area fabrication—that was adapted and simplified from the FPD fabrication process. The fabrication processes remain the same as those specified in Section 4.1, except for the photoresist coating and metal deposition, which are replaced by spray-coating and magnetron sputtering processes due to the large substrate size, respectively. The large-area UV exposure was achieved using an in-house-developed and custom-built large-area mask aligner system. All other fabrication equipment, except for the PECVD and magnetron sputtering process, were in-house-developed and custom-built. This array area represents the largest actuatable MEMS device to date, to the best of our knowledge. Its fabrication proves its scalability and the feasibility of mass production.

The large-area smart glass in Figure 7 has 4 × 7 different subfields. A subfield is a subgroup of neighbored micromirrors inside the array that can be separately addressed. This is achieved by dividing a single module into distinct subfields with pre-structured joints and connection lines that are small enough to be invisible to the naked eye. All 28 subfields can be addressed individually and separately. They are all actuatable at 75 V (fully closed state), with a reaction time of about 0.1 ms. Detailed characterization work is still ongoing and shall be published separately. Figure 7d shows the addressing of subfield No. 10 into the fully closed state, giving the impression of a dark square blocking the view through the glazing. Moreover, combinations of several subfields can be actuated at the same time in an identical way. Subfield addressing is necessary to implement personalized light steering, as shown in Figure 3b.

Industrial manufacturing technologies such as the process automation and optimal control of the fabrication environment can increase the yield and reduce the cost per unit area of micromirror. Even though the operating voltage of a micromirror is independent of the area it covers, the total current is influenced by the area. Therefore, along with the electrode resistivities and dielectric properties of the isolation layer, the area of the micromirrors affects the actuation characteristics. However, this can also be solved by implementing subfields with a separate addressing option. In summary, the feasibility of scaling-up MEMS smart glass has been proven with the fabrication of 71 × 128 cm^2^ in addition to the previously achieved 30 × 30 cm^2^ [25] MEMS smart window modules. Nonetheless, research work in this direction is still ongoing, particularly in the direction of fabrication process optimization to improve overall yield. Regarding ongoing research on the improvement in the clarity of the view through the modules, refer to Donatiello et al. [55].

### 4.5. Power Consumption of Switching and Holding Statuses

**Figure 8:**
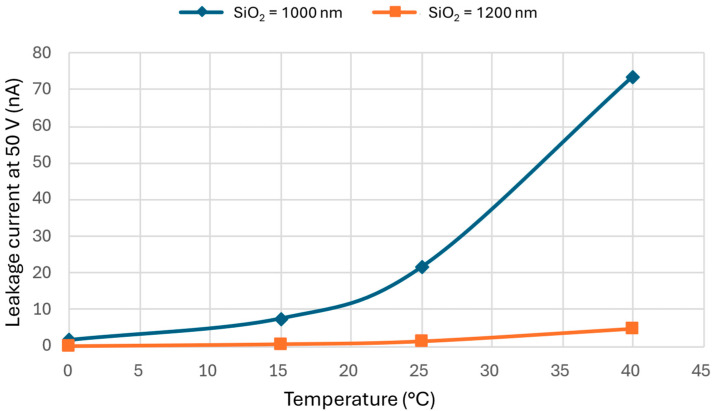
Leakage current through SiO_2_ insulation layers (1000 nm and 1200 nm) measured at 50 V as a function of temperature.

In MEMS smart glass, the power consumption in the holding state is primarily influenced by the leaked current through the SiO_2_ insulation layer. Leaked current measurements were conducted for a 0.0025 m^2^ sample over a temperature range of 0–40 °C. Figure 8 shows the leakage of current measured at 50 V for two samples with different SiO_2_ thicknesses at four different temperatures. The graph shows that the leakage of current increases with rising temperature, indicating that the power consumption of MEMS smart glass also rises as temperature increases. This trend can be explained by the temperature dependence of various bulk conduction mechanisms in insulating materials, including Poole–Frenkel, trap-assisted tunneling, hopping conduction, space-charge-limited conduction, and impurity conduction, as well as the electrode-limited conduction mechanisms like Schottky emission, Fowler–Nordheim tunneling, direct tunneling, and thermionic field emission.

At 20 °C with an applied voltage of 40 V, the power consumption was 0.018 mW/m^2^ for 1.2 μm SiO_2_ and 0.3 mW/m^2^ for 1 μm SiO_2_. As a comparison, the power consumption for smart SPD glass was reported to be independent of temperature [56], while for PDLC, the power consumption decreases as temperature rises [57,58].

Figure 8 depicts leaked current measurements through SiO_2_ layers of 1 µm and 1.2 µm thicknesses. This measurement is very important in determining the energy consumption of MEMS smart glass that utilizes said material as the insulating layer. Insulators have a complex dependence on the applied voltage and temperature. This is due to the participation of a large number of (i) bulk conduction mechanisms: trap-assisted tunneling, Poole–Frenkel, hopping conduction, impurity conduction, and space-charge-limited conduction; and (ii) electrode-related mechanisms such as Fowler–Nordheim tunneling, direct tunneling, Schottky emission, and thermionic field emission. Insulators at room temperature and above show a current increase with rising temperatures (the same trend like in semiconductors, albeit due to different mechanisms). In addition, thicker insulating SiO_2_ has a lower leakage current, as expected, although this also results in higher actuation voltage due to the increased distance between the electrodes.

In MEMS smart micromirror glass, energy is consumed in two states, namely, in the holding state, in which the micromirrors are being held at a specific angle, and in the actuation state, where the shifting of the charges in the electrodes takes place. In most cases, the holding current, i.e., the leakage of current, is the largest contributor to power consumption. Since the window experiences different temperatures during day and night as well as weather and seasonal changes, the energy consumption not only depends on the user scenarios but also on the temperature.

For MEMS smart micromirror glass, no power is consumed when the window is fully open. However, various actuation voltages are needed for different opening angles to achieve active light steering with micromirrors, which means that the power consumption during the holding mode depends on the opening angle of the micromirrors. When the windows need to be completely closed at night for privacy or to prevent heat loss in winter, the power consumption is about 0.3 mW/m^2^ at 50 V. In summer, fully closed windows require approximately 1.5 mW/m^2^ to maintain a comfortable indoor temperature. The power consumption in holding modes ranges between 0 and 1.5 mW/m^2^, with an average estimated as 0.8 mW/m² throughout the year.

Information on the power consumption of other smart glass technologies is necessary to establish a comparison with MEMS smart micromirror-glass. Thorough searches of the scientific literature and available datasheets were conducted with focus on the existing commercially available technologies, namely, PDLC [57,58,59,60,61,62,63,64,65,66,67,68,69,70,71,72], SPD [56,73,74,75,76,77,78], and electrochromic [79,80,81,82,83,84,85] smart glass. Concerning power consumption, some of the values reported by different research groups and company homepages vary considerably. In most cases, technical details are not given or are incomplete, so it was impossible to draw a fair comparison or conclusions. To obtain at least a very rough value, all available data were taken and averaged. The large number of citations provides the number of values taken for the average: 16 for PDLC, 7 for SPD, and 7 for electrochromic smart glass. The average power consumption for smart electrochromic glass in holding mode is almost zero, while the average power consumption in switching mode is about 1 W/m^2^. For smart PDLC glass, the average power consumption in holding mode is about 5 W/m^2^, and the switching mode consumes about 5 W/m^2^ on average. Smart SPD glass also consumes on average 3 W/m^2^ in both holding and switching modes.

### 4.6. Reliability Studies Using Different Rapid-Aging Tests

Windows face harsh environmental conditions, including temperature fluctuations; ultraviolet (UV), visible, and infrared (IR) radiation; mechanical vibrations; and shocks; as well as weather factors like snow, ice, hail, rain, humidity, wind, and sandstorms. Rapid-aging tests are conducted to gather extensive information about potential failures, allowing us to estimate mean time-to-failure (MTTF) rates, lifetimes, and reliability data. These tests reveal failure probability over time (spanning several years) in the form of the Weibull or bathtub curve [86], which shows a decreasing failure rate initially, followed by a prolonged period of low failure rates, and ending with an increase in failure rates.

**Figure 9:**
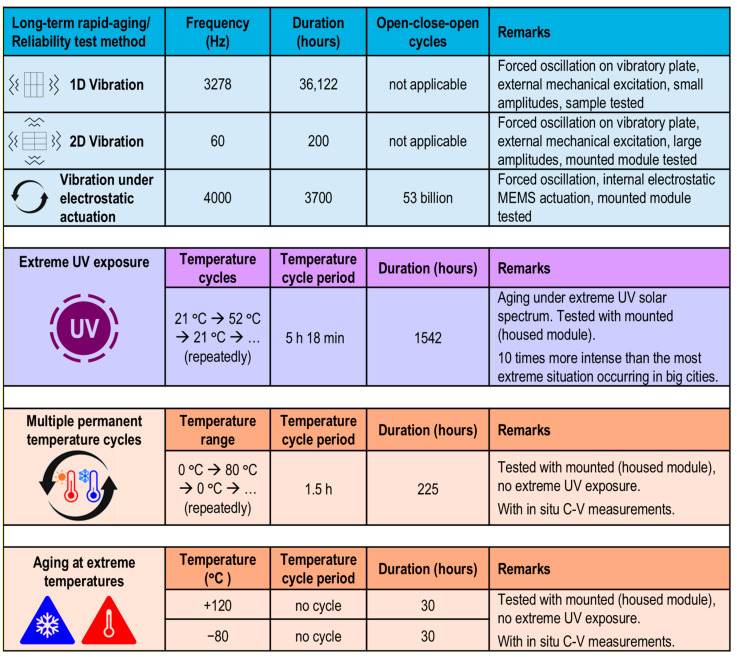
Recent results from various reliability studies and rapid-aging tests conducted for MEMS micromirror arrays.

The micromirror arrays for active light steering in smart windows are sealed and protected in a noble gas atmosphere within insulated glazing. This arrangement greatly benefits the micromirror arrays by eliminating the moisture and oxygen that could damage the metallic surfaces of the mirrors over time. Consequently, the remaining reliability concerns are primarily related to mechanical shock, vibration, repeated movement, extreme temperature, sudden temperature changes, and UV radiation. Comprehensive experiments addressing these issues are ongoing, with the promising preliminary results being summarized in Figure 9. In almost all instances, MEMS micromirror arrays in double-insulated glazing have been used for testing. The only exception was the 1D vibration test, which was conducted using MEMS arrays on 10 × 10 cm^2^ glass substrates that were not housed in modules, as weight reduction was necessary to achieve high vibration frequencies (>3 kHz).

To experimentally simulate rapid aging, vibration tests using one-dimensional (1D) and two-dimensional (2D) mechanical vibration, along with electrically actuated amplitude modulation response studies, were conducted. For the long-term 1D vibration test, micromirror samples were placed in a horizontal position and underwent forced oscillation under external sinusoidal vertical mechanical excitation. The micromirrors survived after mechanical excitation with frequency variation in the range of 0–6 kHz for more than 36,000 h.

For the 2D vibration tests, two electromotors with unbalanced axes were utilized. The micromirror samples were positioned horizontally and subjected to forced oscillations under sinusoidal lateral mechanical excitation at frequencies up to 100 Hz. The two masses could be adjusted to create variable phase shifts (0–2π) between them. A phase shift of π/2 produced the maximum tilt vibration, while a phase shift of π yielded minimal effects. In contrast, phase shifts of 2π or 0 resulted in maximum acceleration values. Several samples were subjected to long-term vibration testing at a frequency of 60 Hz under maximum acceleration conditions (phase shift of 2π for the two masses). These tests simulated the building vibrations generated by compressors and pumps. The survival of the micromirrors after this test showed that no failure of MEMS smart micromirror-glass is expected during the transportation and installation process or during long-term use.

Measuring the lifetime of micromirrors involves actuating them at high frequencies. By exposing micromirrors to a high-frequency excitation signal, many on/off cycles can be generated in a short period. Since most mechanical failures are directly linked to the number of operational cycles, high-frequency actuation can accelerate the occurrence of these failures. This approach enables a more accurate assessment of the micromirrors’ lifetime.

**Figure 10:**
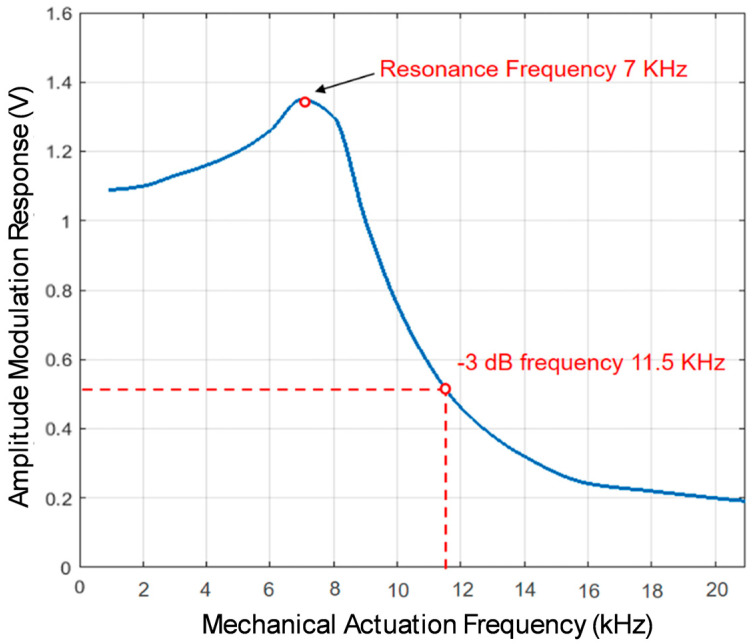
Amplitude modulation response (AMR) curve for MEMS micromirror.

The AMR (amplitude modulation response) of micromirror arrays is determined by varying the excitation signal frequency and recording the peak amplitudes of the micromirrors’ responses, as shown in Figure 10. Since the electrostatic attraction of the micromirrors occurs during both the positive and negative half-cycles, the micromirrors respond to the excitation signal at twice the frequency of the original excitation signal. The resonance frequency, identified at the peak voltage of the AMR curve, was measured to be 7 kHz. Considering the typical average changes in a micromirror’s state in smart windows initiated by user interactions, the over 53 billion cycles of opening and closing movements performed at a 4 kHz excitation frequency would approximately correspond to angle adaptations for an extrapolated lifetime of at least 70 years. This measurement was performed at room temperature.

To test the performance of micromirrors under strong UV irradiation, a sample was placed in a chamber insulated with expanded polystyrene foam (EPS), and the inner walls of the chamber were coated with ultra-high-reflective UV foils to maximize the use of UV light. Solar irradiance in the UV spectrum was simulated using UV LED arrays that emitted a given range of wavelengths. The spectral irradiance of the LEDs was selected to be approximately ten times higher than what is typically found in extreme urban environments. UV LEDs also served as efficient heat sources and were utilized to conduct temperature cycling simultaneously. Each temporal cycle consisted of a radiation phase, followed by a dark phase, during which the temperature varied from 21 °C to 52 °C. To verify the functionality of the MEMS array throughout the test, C-V measurements were performed both in situ and during the breaks when the temperature returned back to room temperature. Also, in this test, no defective micromirrors were observed.

In another test, temperature cycles (between 0 °C and 80 °C) were conducted to simulate varying weather conditions. Multiple cycles were executed, with each cycle having a 20 min heating period, a 20 min holding period at 80 °C, a 20 min active cooling period, and a 30 min holding period at 0 °C. C-V measurements were performed to ensure that the MEMS smart glass module remained fully functional throughout the tests. The micromirror arrays also demonstrated complete operability even at extreme temperatures of −80 °C and 120 °C. However, lower and higher temperatures beyond this range could not be tested due to the limitations of the climate chamber, which could not accommodate more extreme conditions.

Additionally, the high-reliability and user-friendliness needs also require smart glass to be sufficiently transparent for the microwaves used in mobile and data communication systems based on RF frequencies that involve data transfer. Therefore, preliminary measurements were performed through a 200 × 200 cm^2^ steel wall furnished with absorber material, acting on the magnetic field of the electromagnetic wave. The wall had an opening of 29 × 29 cm^2^. The measurements were performed at 2.1 GHz. Transmission measurements were conducted (i) without a sample, (ii) with float glass coated with FTO, and (iii) with float glass containing FTO and MEMS micromirrors. The resulting measured values of these three cases were (i) –28.29 dB, (ii) –33.83 dB, and (iii) –33.84 dB, respectively. This preliminary result is very promising since the FTO provides the largest absorption. Additionally, FTO or other types of TCOs are used in all other smart glass technologies. Most importantly, the MEMS micromirror arrays absorbed nearly nothing in these preliminary experiments.

## 5. Simulation of Energy Savings

The primary objective of MEMS smart windows is to maximize the utilization of daylight while reducing the energy consumption associated with heating, cooling, and artificial lighting, thus contributing to huge annual energy savings. Consequently, window orientation plays a crucial role, which is influenced by geographical location and seasonal variations in solar positioning. In the northern hemisphere, windows should ideally be south-oriented to access the maximum amount of solar radiation and conversely north-oriented in the southern hemisphere. Seasonal sun paths differ significantly: during summer, the sun rises in the northeast and sets in the northwest, following a high and prolonged trajectory at solar noon near the summer solstice. The tilt of the northern hemisphere toward the sun extends daylight duration and increases solar intensity, resulting in higher ambient temperatures. Conversely, in winter, the sun rises in the southeast and sets in the southwest, maintaining a lower and shorter path in the sky. During the winter solstice, the northern hemisphere tilts away from the sun, resulting in shorter daylight hours and reduced heating. Throughout the year, the sun path remains in between these two extreme positions, with solar radiation changing according to seasonal shifts.

The simulation of energy savings was carried out for different window orientations, types of low-e coatings, weather databases, and engine class reductions for MEMS smart windows in comparison with conventional windows with tiltable aluminum blinds. A model office room on the sixth floor in Kassel, Germany, located at 51.18° N latitude, 231 m above sea level, and in the central area of low northern, western, and eastern mountain ranges (up to 600 m above sea level), was considered. The office room dimensions were a 12 m length, a 8.3 m width, and a 3 m height. Its window area was specified as triple-insulation windowpane (12 m in length and 3 m in height), covering the whole south-facing wall. The plain office room was occupied by 20 persons and furnished with 10 tables, 20 chairs, 20 computers, 20 mobile phones, and 10 cupboards. For the simulation, it was assumed that all occupants engaged in identical activities during working hours consistently throughout the year. Due to the energy-saving capacity of MEMS smart windows, which is achieved by enabling the improved distribution of daylight and solar radiation, the engine class for the lighting, heater, and air conditioner was not required to be as large as for windows with aluminum blinds. Therefore, the engine class for the MEMS smart windows was reduced by 15% for the lighting, heater, and air conditioner compared to the windows equipped with aluminum blinds.

**Figure 11:**
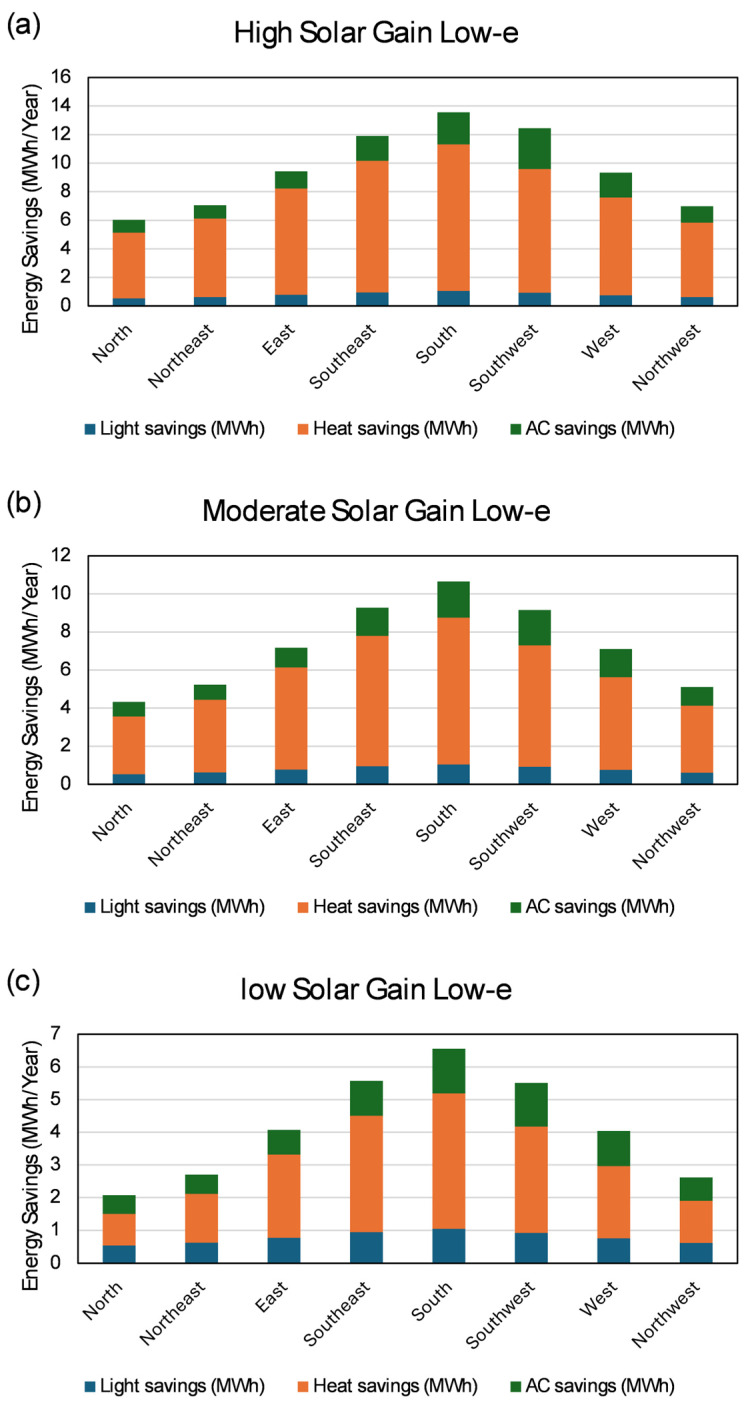
Overall yearly energy savings (light, heat, and AC) for a model room equipped with MEMS smart windows having (**a**) high, (**b**) moderate, and (**c**) low solar gain, low-e coating for different window orientations in Kassel, Germany.

A simulation tool, “*SAVINGS*” [27], was developed for the calculation of energy savings. Three different types of low-e coatings—high, moderate, and low solar gain—were considered, and their impact on energy savings was calculated (Figure 11). The effect of low-e coatings on light saving was insignificant due to the nearly identical visible light transmission; however, it had a strong influence on yearly air conditioning (AC) and heat savings due to the different infrared transmission characteristics.

A test reference year (TRY) database was used in the calculation. This included a combination of hourly data for Kassel over the year from 1 January to 31 December recorded by the “Deutscher Wetterdienst” (German Metrological Service) over a considerably long observation period of 1995–2012 [87,88]. Meteorological parameters such as cloud coverage, wind velocity, temperature, heat irradiance, visible irradiance, and so on are permanently recorded. An average value was saved each hour for all the parameters, which is henceforth referred to as hourly data. The methodology of the data construction in the TRY database over the long period of time is described in EN ISO 15927–4:2005 [89] The detailed calculation methodology considering all the associated solar angles, total incident irradiance, window parameters (U_g_ and g values), different low-e coatings, window orientations, light transmissions (Fresnel equations), as well as the different conditions in different seasons and time of the day are reported in [27].

For south-oriented MEMS smart windows, the annual light, AC, and heat savings for a high-solar-gain, low-e coating were calculated to be around 1 MWh, 2.2 MWh, and 10.2 MWh, respectively. Analogously, the same calculation for a moderate solar gain resulted in values of 1 MWh, 2 MWh, and 7.7 MWh, respectively. Likewise, for the low-solar-gain, low-e coating, the calculated amounts were 1 MWh, 1.3 MWh, and 4.1 MWh, respectively.

The calculation also revealed that the yearly energy savings of the south-oriented MEMS smart windows with high-, moderate-, and low-solar-gain low-e coating were 13.5 MWh, 11 MWh, and 6.5 MWh, respectively. The availability of solar radiation varies with window orientation from south to north, which results in quick or slow energy savings.

For high solar gain and low-e, the annual light, AC, and heat savings were calculated to be 1 MWh, 2.2 MWh, and 10.2 MWh, respectively, for south-oriented windows, which decreased to the minimum (0.5 MWh, 1 MWh, and 4.6 MWh, respectively) when changing the window orientation toward the north with a deviation of approximately 55%. Moreover, a detailed calculation and analysis of the effect of investment and other parameters like the U_g_ and g values can be found in [27]. The simulation was extended to other cities (Kassel, Winterberg, Flensburg, and Konstanz in Germany), as well as global cities like Qingdao, China, and Riyadh, Saudi Arabia.

## 6. Conclusions

Electrostatically actuatable MEMS micromirror arrays fabricated on a transparent substrate are appealing for both transmission and reflection applications. They exhibit low power consumption, fast switching speed, and significant energy saving potential, and they are cost-effective with high mechanical stability due to their miniaturization, leading to a long lifetime. The dimensions and array sizes of metallic MEMS micromirrors or microshutters vary significantly depending on their intended application. They have applications in daily optics, such as smart windows for personalized daylight steering, active laser safety goggles, and ring shutters [40] for endoscopy, spectroscopy, and more.

The development of planarized micromirror arrays makes the MEMS smart window technically viable for active light steering. The electrostatic actuation voltage and switching time of a micromirror depend on the micromirror array dimensions. The following parameters were obtained in different samples: the actuation voltage can be as low as 12 V, and the fastest closing time measured for a MEMS microshutter was 1 μs, which is significantly faster than the times reported for all other smart glass window technologies. Additionally, a modulation contrast of 7700 for light intensity was reported for MEMS smart glass, significantly surpassing the modulation contrasts noted for all other smart glass technologies. Moreover, in the event of an abrupt power outage, both PDLC and SPD glazing can remain opaque, which poses a safety risk by obstructing visibility from the outside during emergencies. In contrast, MEMS smart glazing fully meets safety requirements in such situations since it is by default in the open state.

Micromirror arrays in smart windows have been developed with the aim of active daylight steering irrespective of season, sun position, or user movement inside the room. Generally, micromirrors can be actuated only in one direction—toward the substrate from an open position (90° to the substrate), generating only tilt angles (Φ). This limits the light steering capacity when the sun position is low in the sky (e.g., early morning and late afternoon for a south-oriented window) and in the case where the user moves sideways. Therefore, in another work, a flexible torsion-like hinge was integrated into the existing micromirror design to achieve two degrees of freedom (tilt angle Φ and torsion angle θ), allowing daylight steering throughout the day independent of the sun’s position in the sky or user movement inside the room [24,26].

Electrochromic technologies currently provide the best results in providing a clear view through the window among the smart window technologies. Another research work is ongoing to minimize the diffraction effects and provide a clearer view through MEMS smart glass by utilizing irregular micromirror shapes (non-periodic apertures) within the arrays [18,55]. Furthermore, an ultra-thin germanium (Ge) layer can be coated to alter the color of metallic micromirrors and minimize surface glare effects [25]. The successful fabrication of micromirrors on flexible substrates makes it promising for applications like car sunroofs and augmented reality [90]. These are important milestones in the research progress of micromirror and microshutter arrays, along with the resulting patents in [91,92,93] as well as the recent applications in Section 7.

Despite their limitations, smart window technologies have the potential to provide significant energy savings by utilizing daylight and solar heat. In holding mode, the power consumption is the lowest for electrochromic and MEMS technologies, moderate for SPD, and the highest for PDLC. In switching mode, MEMS has the lowest power consumption, followed by electrochromic with low consumption, SPD with moderate consumption, and PDLC with the highest consumption.

The energy-saving capacity of MEMS smart windows was presented for a model office room in comparison to conventional aluminum-blind-equipped windows for a location in Kassel, Germany, where different scenarios were considered with different combinations of window orientation, the estimated price of micromirror array glazing, U_g_ values, and g values, as well as engine power for heating, lighting, and AC. The low-e coating types and window orientation have a strong influence on annual energy savings. The load requirement for modern lighting, heating, and AC equipment can be significantly reduced for buildings equipped with MEMS smart windows, whereas the power consumption trend remains the same in buildings with conventional windows. Although MEMS smart windows require more initial investment, the energy saving begins immediately from the start and gradually becomes significant over time. Annual energy savings of 13.5 MWh were calculated for the best-case scenario for a south-oriented window with a high-solar-gain and low-e coating and in the worst case, 2 MWh, for the north-oriented window with a low-solar-gain low-e coating. If a meteorological database is available, the simulation tool *“SAVINGS”* can be used for all cities in the world by simply uploading the dataset. The simulation shows the potential to reduce CO_2_ emissions by enormous amounts using MEMS smart windows via efficient heat management and light steering in buildings. Moreover, only 1 g of aluminum is required to produce a 1 m^2^ MEMS smart window, which is approximately 1000 times less than that of aluminum blinds.

Micromirrors offer rapid switching, no unintentional tinting, low power consumption, better solar control, and area-selective functionality. Factors such as stability in chemical environments (humidity, oxygen), resistance to temperature cycling, and durability against UV exposure are essential for the long-term performance and reliability of smart window technologies. Various long-term rapid-aging and reliability tests on micromirrors demonstrate their stability across a temperature range from −80 °C to 120 °C, with a lifetime of over 70 years.

To limit global warming and CO_2_ emissions, smart glass in buildings is a very important technology for the future. MEMS smart glass is found to be superior to other smart glass technologies on the market. MEMS smart glass opens new and attractive perspectives for huge energy savings and modern concepts for the heat and energy management of buildings. The upscaling of MEM smart glass from nearly 1 m^2^ to even larger areas seem to be possible from the current point of view. Regulations and policies to increase energy savings in buildings, as well as increasing the awareness of building owners of the recent issues caused by energy crisis, have created a demand to be filled. Particularly for high-rise buildings with more than seven floors, MEMS smart glass has a unique selling point as a sun-shading and daylighting system.

## 7. Patents

1. H. Hillmer, G. Xu: Lichttechnisches Modul für eine Gebäudefassade, EP 4102024 B1 (2024).
2. H. Hillmer, Spiegel-Shutter-Array, EU 3964877 (2024).

## Data Availability

Not applicable.
